# The Effect of Tianmai Xiaoke Pian on Insulin Resistance through PI3-K/AKT Signal Pathway

**DOI:** 10.1155/2016/9261259

**Published:** 2015-11-10

**Authors:** Nana Wang, Tiegang Li, Ping Han

**Affiliations:** ^1^Endocrinology Department, Shengjing Hospital of China Medical University, Shenyang 110004, China; ^2^Emergency Department, Shengjing Hospital of China Medical University, Shenyang 110004, China

## Abstract

In the clinical setting, given the potential adverse effects of thiazolidinediones and biguanides, we often have difficulty in treatment that no other insulin sensitizers are available for use in type 2 diabetic mellitus (T2DM) patients. Tianmai Xiaoke Pian (TMXKP) is a traditional Chinese medicine tablet, which is comprised of chromium picolinate, Tianhuafen, Maidong, and Wuweizi. To understand its mechanism of action on insulin resistance, TMXKP (50 mg/kg orally) was tested in T2DM rats (induced by a high-fat diet and streptozotocin). Eight weeks later, fasting blood glucose (FBG) and oral glucose tolerance tests (OGTT) were performed. Area under the curve (AUC) and homeostatic model assessment of insulin resistance (HOMA-IR) were calculated, and PI3-K/AKT signal pathway-related genes and proteins were tested by reverse transcription-polymerase chain reaction (RT-PCR) and western blot analysis in muscle, adipose, and liver tissues, respectively. TMXKP significantly reduced FBG, OGTT, AUC, and HOMA-IR in diabetic rats (*P* < 0.05). Furthermore, we also observed that TMXKP could significantly decrease *IRS-1*, *IRS-2*, *PI3-K p85α*, and *AKT2* gene expression and also IRS-1, IRS-2, PI3-K, AKT2, and p-AKT2 protein expression levels (*P* < 0.05) in diabetic rats. These findings confirm that TMXKP can alleviate insulin resistance in T2DM rats through the PI3K/AKT pathway. Thus TMXKP appears to be a promising insulin sensitizer.

## 1. Introduction

Type 2 diabetes mellitus (T2DM) is a chronic metabolic syndrome with an increasing prevalence throughout the world. The most recent data report 37.1 million T2DM patients worldwide; the percentage of these T2DM patients in China is 11.6% and 11.3% in the United States [1, 2]. The main etiology of T2DM is insulin resistance (IR). IR is a physiological condition in which cells fail to respond adequately to the normal actions of the hormone insulin. The body may continue to produce insulin, but the cells in the body become resistant to its actions and, therefore, are unable to use it as effectively, leading to hyperglycemia [3]. To reduce IR, there are several insulin sensitizers that are commonly used, including thiazolidinediones (e.g., rosiglitazone and pioglitazone) and biguanides (e.g., metformin). These agents can improve IR and increase peripheral utilization of insulin, resulting in a decrease in blood glucose.

Thiazolidinediones carry a risk of bone fractures, bladder cancer, and, of special concern, cardiovascular side effects [4]. Although some studies have shown that thiazolidinediones have no additional risks for myocardial infarction or cardiac death [5, 6], caution in its use has been recommended by the U.S. Food and Drug Administration. Because of safety concerns, thiazolidinediones use has been stopped in some areas. The biguanides remain for use in the treatment of IR, and these agents may have gastrointestinal side effects. However, in the clinical setting, we often face the difficulty of having no effective insulin sensitizers to use in certain T2DM patients, especially those who cannot tolerate biguanides.

Tianmai Xiaoke Pian or TMXKP (Hebei Fuge Pharmacy Co., Ltd) is a traditional Chinese medicine tablet, comprised of chromium picolinate (1.6 mg per tablet, 200 *μ*g elemental chromium), Tianhuafen (Radix Trichosanthis, snake gourd root), Maidong (Radix Ophiopogonis, dwarf lily turf tuber), and Wuweizi (Fructus Schisandrae Chinensis, Chinese magnoliavine fruit) [7]. Chromium picolinate is one of the main components of TMXKP. In previous studies, TMXKP was shown to decrease serum glucose and glycated hemoglobin (HbA1c) in T2DM patients and also in rats [7–9]. However, the underlying mechanism of its antidiabetic effects remains unclear. Therefore, in this present study, we examined the effects of TMXKP on insulin downstream signaling PI3-K/AKT pathway, an important insulin pathway, in rats with T2DM.

## 2. Materials and Methods

### 2.1. Animals

SPF grade male Wistar rats (weighing 250 to 300 g) were obtained from the Laboratory Animal Investigation Center of China Medical University (Liaoning, China). The rats were housed in SPF facilities at the Animal Center of Shengjing Hospital of China Medical University, under a 12-hour dark-light cycle and fed ad libitum during the experiment. The animal protocols used were approved by the Institutional Animal Care and Use Committee at Shengjing Hospital of China Medical University. All animal experiments were carried out in accordance with the National Research Council Guide for the Care and Use of Laboratory Animals.

Based on previous articles about the T2DM model [10–12] and our preliminary experiment, a T2DM model was induced by high-fat diet and streptozotocin (STZ) injections. Rats were fed with a high-fat diet (22% fat, 48% carbohydrates, and 20% protein, 20.08 kJ/g) [13, 14] for 4 weeks prior to intraperitoneal injection of a single 25 mg/kg dose of STZ (dissolved in 0.05 M citrate buffer at pH 4.5, prepared immediately before use). Two weeks after the STZ injection, rats were assessed by fasting blood glucose with blood collected from the tail vein. T2DM was diagnosed as a fasting blood glucose ≥7.8 mmol/L on two occasions or a nonfasting blood glucose ≥11.1 mmol/L; rats induced with T2DM were considered as diabetic rats and were selected for subsequent experiments. Diabetic rats were fed with the same high-fat diet throughout the experiment.

### 2.2. Experiment Design

Diabetic rats were randomly divided into two groups: a diabetic group (*n* = 10) and a TMXKP-treated group (*n* = 10, given 50 mg/kg TMXKP intragastrically daily). Weight-matched normal rats were selected as the control group (*n* = 10). Based on the recommended dose of TMXKP for humans (0.48 g/d), a corresponding dose for rats was estimated at 50 mg/kg. The TMXKP-treated group received 0.48 g TMXKP dissolved in 2 mL 0.9% saline; the control group received an equal volume 0.9% saline without TMXKP. All diabetic rats received a high-fat diet during the experiment, while nondiabetic rats in the control group were fed a normal diet (5% fat, 53% carbohydrate, and 23% protein, 14.6 kJ/g). After 2 months, all rats were anesthetized with sodium pentobarbital and then sacrificed. Blood, left gastrocnemius muscle, greater omentum adipose tissue, and liver were removed immediately for further analysis.

### 2.3. Fasting Blood Glucose and Oral Glucose Tolerance Test

Fasting blood glucose and oral glucose tolerance tests (OGTT) were performed every 2 weeks after diagnosis of diabetes using blood samples taken from the tail vein. Generally speaking, following a 12-hour overnight fast, blood from the tail vein was collected at 0, 30, 60 and 120 minutes after glucose lavage (at 1 g/kg of body weight). The area under the curve (AUC) was calculated for blood glucose (BG) from the OGTT as follows: AUC = 1/4 × (BG_0_) + 1/2 × (BG_30_) + 3/4 × (BG_60_) + 1/2 × (BG_120_) [15]. Blood glucose was measured using an Accu-check Performa Nano glucose meter (Roche, Germany), and insulin was detected by enzyme-linked immunoassay (ELISA; rat insulin ELISA kit, Thermo Scientific) in accordance with the method of Kekow et al. [16]. Homeostatic model assessment of insulin resistance (HOMA-IR) was calculated as [17]:

(1) $$\begin{array}{c} \text{HOMA-IR}=\frac{\text{FBG }\left(mmol/L\right)\times \text{FINS }\left(\mu U/mL\right)}{22.5}, \end{array}$$

where FBG is fasting blood glucose and FINS is fasting insulin concentration.

### 2.4. Reverse Transcription-PCR Analysis

The total RNA of skeletal muscle, liver, and adipose tissues from rats was extracted using the Trizol method, according to manufacturer's instructions.* IRS-1*,* IRS-2*,* PI3-Kp85α*,* AKT2*, and *β-actin* gene expression was detected by reverse transcription-polymerase chain reaction (RT-PCR) analysis. Primer sequences and PCR conditions are listed in Table 1. The relative quantification results of gene expression were normalized on *β*-actin transcript levels. Results were expressed as the mean of three independent experiments performed in triplicate.

### 2.5. Western Blot Analysis

Liver, adipose tissue, and skeletal muscle lysates were homogenized by centrifuging at 15,000 r/min for 30 minutes at 4°C. Tissue homogenates were subjected to 10% SDS-PAGE and transferred to nitrocellulose membranes. Nitrocellulose membranes were incubated at 4°C overnight with primary antibodies against IRS-1, IRS-2, PI3-K, AKT2, p-AKT2, and *β*-actin, respectively. Immunoreactive proteins were visualized using enhanced chemiluminescence western blotting detection reagents and detected by HMIAS-2000 Imaging System. Band densities were determined by BioRad Quantity One software and quantified as the ratio to *β*-actin.

### 2.6. Statistical Analysis

The results are presented as mean ± standard deviation. Statistical significance was determined by one-way analysis of variance (ANOVA) followed by Dunnett *C* test for unequal variances data. For data with equal variances assumed, ANOVA was followed by least squared differences (LSD) test. SPSS16.0 software was used. *P* < 0.05 was considered as statistically significant.

## 3. Result

### 3.1. General Conditions

At the beginning and end of the experiment, weight, total cholesterol, and triglycerides for all rats were recorded. As shown in Table 2, we could observe an obvious decrease in body weight in diabetic rats compared with normal (control group) rats (*P* < 0.05). And there was no difference in body weight between the TMXKP-treated and untreated diabetic groups. Total cholesterol and triglycerides levels were elevated in the diabetic group compared with the control group, but TMXKP was expected to decrease these values in the treated diabetic group (*P* < 0.05).

### 3.2. Blood Glucose and Insulin Resistance Index

**Figure 1:**
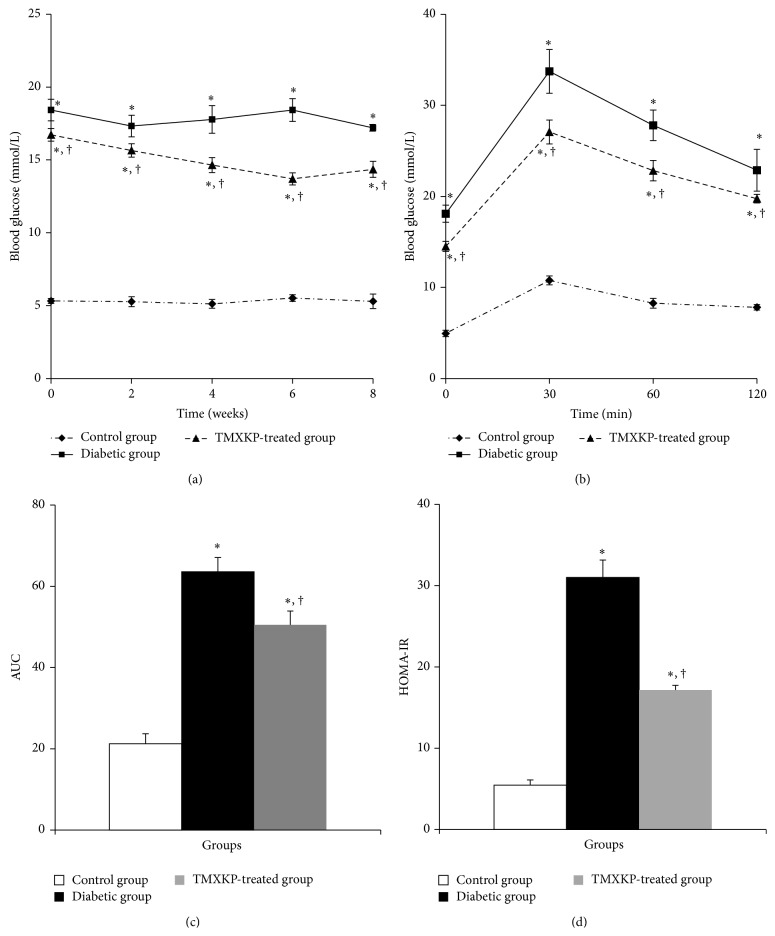
Blood glucose and insulin in the three groups. (a) The fasting blood glucose in three groups at 2 weeks. The fasting blood glucose was measured every 2 weeks in the experimental procedure. There was significant decrease in fasting blood glucose after TMXKP treatment compared with the untreated diabetic group. (b) The OGTT at 8 weeks. OGTT was measured at the end of experiment. We could observe a decrease in glucose at all the time points. (c) The AUC of three groups. The AUC was calculated as 1/4 × (BG_0_) + 1/2 × (BG_30_) + 3/4 × (BG_60_) + 1/2 × (BG_120_). All glucose values were the result of the OGTT. (d) HOMA-IR value of three groups at 8 weeks, which was selected to evaluated insulin resistance. After treatment with TMXKP, insulin resistance was improved.

The fasting blood glucose of diabetic rats was significantly higher than that in the control group at all time points. Fasting blood glucose in the TMXKP-treated group decreased significantly compared with the untreated diabetic group (*P* < 0.05, Figure 1(a)).

For the OGTT test, blood glucose levels in the untreated diabetic group were higher than those of the control group at all time points after oral glucose administration (*P* < 0.05). However, blood glucose after OGTT in the TMXKP-treated group was significantly lower compared with the untreated diabetic group (*P* < 0.05, Figure 1(b)).

From the AUC of the three groups, we could observe an obvious decrease in glucose in the TMXKP-treated group compared with the untreated diabetic group (*P* < 0.05, Figure 1(c)).

To evaluate IR, we calculated HOMA-IR by the formula described above. After comparisons, the HOMA-IR value in TMXKP-treated group was significantly decreased compared with that of the untreated diabetic group (*P* < 0.05, Figure 1(d)). This decrease indicates that TMXKP could reduce IR in treated diabetic rats.

TMXKP is Tianmai Xiaoke Pian; AUC is area under curve, mmol/L*∗*h; OGTT is oral glucose tolerance test; HOMA-IR is homeostatic model assessment of insulin resistance; ^*∗*^ *P* < 0.05, versus control group. There were significant differences when compared with control group; ^†^ *P* < 0.05, versus diabetic group. There were significant differences when compared with the untreated diabetic group.

### 3.3. Reverse Transcription-Polymerase Chain Reaction Analysis

**Figure 2:**
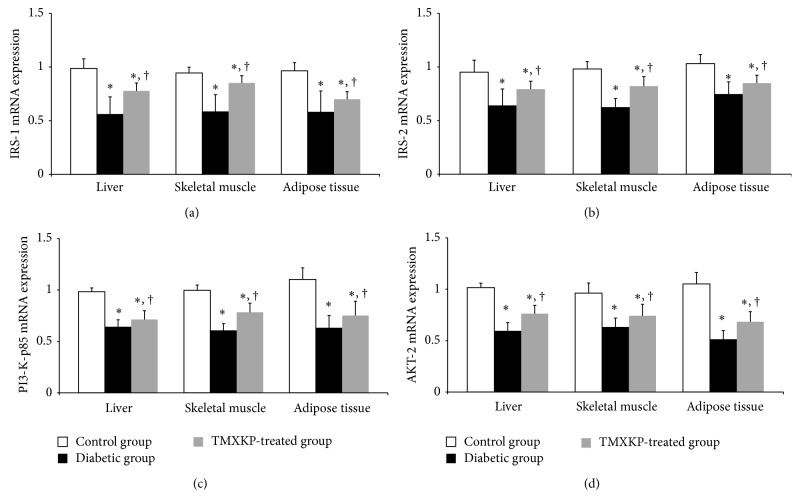
PI3-K/AKT signal pathway-related gene mRNA expression levels in different tissues.* IRS-1*,* IRS-2*,* PI3-K p85α*, and* p-AKT2* mRNA expression were tested using RT-PCR in liver, skeletal muscle, and adipose tissues from rats of each experimental group. RT-PCR: reverse transcription-polymerase chain reaction; TMXKP: Tianmai Xiaoke Pian. Data are expressed as means ± SD (*n* = 10). ^*∗*^ *P* < 0.05 versus control group; ^†^ *P* < 0.05 versus untreated diabetic group.

PI3-K/AKT signal pathway is thought to be an important signal pathway in IR. To detect the effects of TMXKP on IR, liver, left gastrocnemius muscle, and greater omentum adipose tissues were removed. PI3-K/AKT signal pathway-related gene expression levels were detected by RT-PCR analysis, including IRS-1, IRS-2, PI3-K p85*α*, and AKT2 genes. The primers used are listed in Table 1. In the untreated diabetic group, all gene expression levels were significantly decreased compared with the control group (*P* < 0.05). And after TMXKP treatment, gene expression levels were increased compared with the untreated diabetic group (*P* < 0.05). All results are shown in Figure 2.

### 3.4. Western Blot Analysis

**Figure 3:**
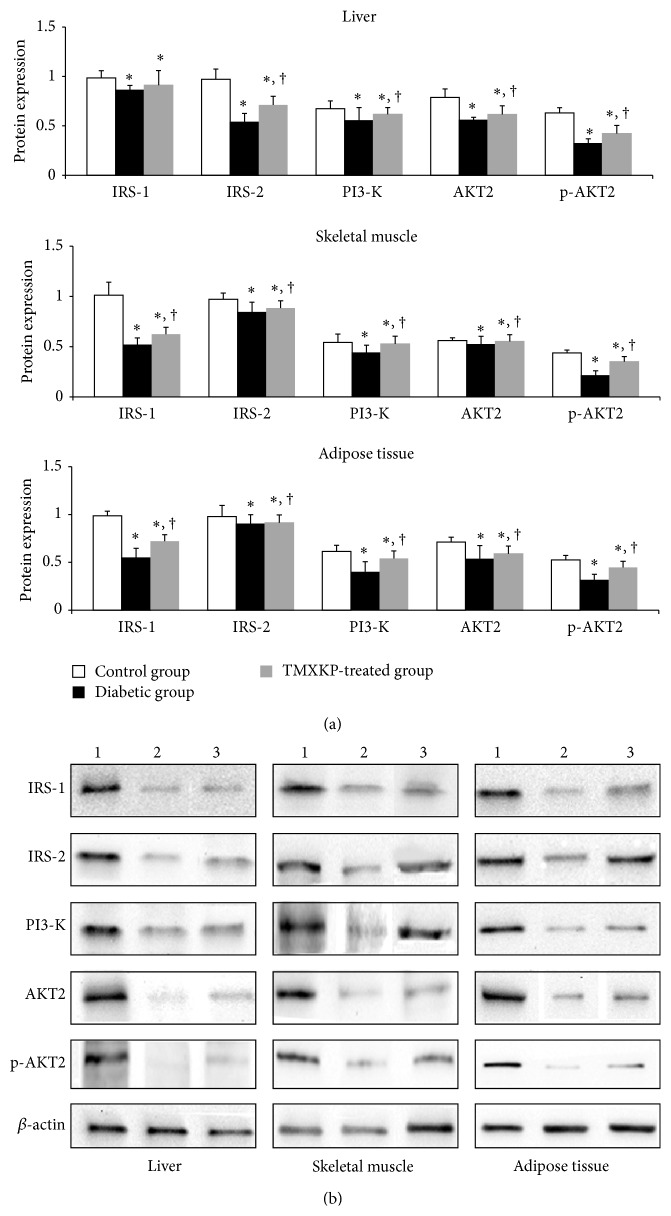
IRS-1, IRS-2, PI3-K, and AKT2 protein expression levels in liver, skeletal muscle, and adipose tissues by western blot. The expression levels of IRS-2, PI3-K, and AKT2 in all tissues were lower in the untreated diabetic group than in controls (*P* < 0.05), while those of the TMXKP-treated group were higher than in the untreated diabetic group (*P* < 0.05). (a) ^*∗*^ *P* < 0.05 versus control group; ^†^ *P* < 0.05 versus untreated diabetic group. (b) 1, control group; 2, untreated diabetic group; 3, TMXKP-treated diabetic group.

IRS-1, IRS-2, PI3-K, and AKT2 protein expression levels were detected by western blot method in liver, skeletal muscle, and adipose tissues. The ratio to *β*-actin was used as the relative expression level of each protein. After comparison, we observed an obvious decrease in protein concentrations in all tissues in the untreated diabetic group compared with the control group (*P* < 0.05, Figure 3). These findings indicated increased IR in diabetic rats compared with controls. After TMXKP treatment for 8 weeks, PI3-K/AKT signal pathway-related proteins were also determined in TMXKP-treated diabetic rats. We observed a significant increase in protein concentrations in all tissues (*P* < 0.05, Figure 3). These findings indicate that IR was reduced by TMXKP through the PI3-K/AKT signal pathway.

## 4. Discussion

T2DM is an increasingly common medical condition that threatens public health. Nearly 1 in 10 of the world's population is affected by T2DM. And a trend for an increasing prevalence is seen year by year. So, the question of how to best fight against T2DM is an important issue for investigators all over the world.

There are two kinds of insulin sensitizers used clinically—thiazolidinediones and biguanides. They both play a crucial role in the treatment of T2DM. However, with the widespread use of these agents, new problems have gradually arisen. Thiazolidinediones have many adverse effects, including the risk of cardiovascular adverse events, bone fractures, bladder cancer, and hepatotoxicity. For this reason, biguanides have become the choice for insulin sensitizers. The major side effects of metformin, a biguanide, are gastrointestinal symptoms (e.g., nausea, vomiting, and diarrhea) and a metallic taste in the mouth [18]. Some patients are intolerant to the gastrointestinal effects of metformin. So there are limitations to the use of biguanides in some patients. As a result, in actual clinical practice, if a patient is intolerant to metformin, there are no other insulin sensitizers available as an alternative.

In this study, to further understand the effects of TMXKP on IR, we tested this agent in rats with STZ-induced T2DM. And the results were in accordance with a previous study by Zhang et al. [7, 9]. After treatment with TMXKP for 8 weeks, characteristic variables of IR including fasting blood glucose, blood glucose following OGTT, AUC of OGTT blood glucose, and HOMA-IR were decreased significantly in TMXKP-treated diabetic rats compared with diabetic rats. Therefore, TMXKP can decrease both blood glucose and IR in diabetes.

As we know, the PI3-K/AKT signal pathway is a classical pathway of insulin in glucose metabolism, having a role in glucose uptake by the liver, skeletal muscles, and adipose tissues [19, 20]. Decreasing or blocking this pathway will reduce the insulin physiological effects that lead to IR.

To investigate the further mechanisms of the effects of TMXKP on IR, we used an RT-PCR analysis method to test the PI3-K/AKT signal pathway-related gene, including* IRS-1*,* IRS-2*,* PI3-K p85α*, and* AKT2* genes in insulin affected tissues: liver, skeletal muscle, and adipose tissues. All gene expression levels were significantly decreased in diabetic rats. After TMXKP treatment, gene expression levels were increased, which indicates alleviators of IR in all insulin-affected tissues. Similar to the regulation of gene expression, we observed increased* IRS-1*,* IRS-2*,* PI3-K p85α*,* AKT2*, and* p-AKT2* expression after TMXKP treatment. This indicated that TMXKP could improve IR through the PI3K/AKT pathway.

Chromium picolinate is one of the main components of TMXKP. The effects of chromium on glucose and insulin are contentious. Many researchers suggest a beneficial effect on glucose intolerance [21, 22]; however, other research has shown it to be ineffective [23, 24]. These findings suggest that although chromium is the main component of TMXKP, perhaps TMXKP does not act exactly in the same manner as chromium. In this experiment, we did not treat animals with chromium picolinate alone but rather with TMXKP. We have shown its beneficial effect on glucose and insulin. But the question remains, what is its active component? Is it chromium picolinate or a metabolic product formed after administration? Additional research is needed to provide this information.

## 5. Conclusions

Because of the issues with the clinical use of insulin sensitizers, we are often faced with a dilemma of choice of treatment. In our investigation, we have shown the anti-IR effects of TMXKP through the PI3K/AKT pathway in detail and have provided evidence for its use. TMXKP may be a promising direction in clinical medicine for the treatment of T2DM.

## Figures and Tables

**Table 1:** Primers of reverse transcription-PCR analysis for genes.

	Primer sequence	Product length
IRS-1-F	TGGACAAACGGAGTAGGG	341 bp
IRS-1-R	CTGGTGGAAG AGGAGGAA	
IRS-2-F	CAAGCAGATCCTGCAGCCACG	196 bp [12]
IRS-2-R	GTTCTCCATAGACAGCTTGGAG	
PI3-K p85*α*-F	ACTGGAGGAAGACTTGAAG	228 bp
PI3-K p85*α*-R	CGTTTCCCAACCATTCGTT	
AKT2-F	ATGTAGACTCTCCAGATGAG	201 bp
AKT2-R	TGAGATAATCGAAGTCATTCA	
*β*-actin-F	GGAAGCTCCGGGAACAAGT	121 bp
*β*-actin-R	TGCCAGCCCATGGATTCTC	

**Table 2:** Baseline characteristics of the three experimental groups.

	Control group		Diabetic group		TMXKP-treated group	
	0 weeks	2 months	0 weeks	2 months	0 weeks	2 months
Weight (g)	276 ± 5	332 ± 7	272 ± 4	286 ± 6^∗^	278 ± 5	287 ± 5^∗^
Cholesterol (mmol/L)	1.78 ± 0.15	1.74 ± 0.20	1.74 ± 0.09	3.72 ± 0.16^∗^	1.69 ± 0.21	3.13 ± 0.15^∗,#^
Triglycerides (mmol/L)	0.68 ± 0.12	0.72 ± 0.12	0.71 ± 0.06	2.39 ± 0.42^∗^	0.68 ± 0.04	1.86 ± 0.13^∗,#^

Weight, total cholesterol, and triglycerides of all rats were recorded at the beginning and at 2 months of the experiment. Weight was decreased in the untreated and TMXKP-treated diabetic groups. We could observe an increase in cholesterol and triglycerides in diabetic rats, but TMXKP could decrease these elevations.

∗, *P* < 0.05, versuscontrol group; #, *P* < 0.05, versus diabetic group.
